# OpenAthens odyssey: challenges of implementing federated authentication for a multi-institutional user population

**DOI:** 10.5195/jmla.2021.1170

**Published:** 2021-10-01

**Authors:** Joanne Romano, Nha Huynh

**Affiliations:** 1 joanne.romano@library.tmc.edu, Head of Resource Management and Discovery Services, The Texas Medical Center Library, Houston, TX; 2 nha.huynh@library.tmc.edu, Systems Librarian, The Texas Medical Center Library, Houston, TX

**Keywords:** OpenAthens, remote authentication, health sciences libraries, system migration, EZproxy

## Abstract

**Background:**

The Texas Medical Center (TMC) is home to one of the world's largest cohorts of faculty, students, researchers, and clinicians who rely on seamless and immediate access to digital biomedical and health resources. This group is served by the TMC Library, with a collection that includes over 380,000 ebooks and 59,000 ejournals. In 2018, the TMC Library implemented OpenAthens, a federated authentication system to replace a locally hosted instance of EZproxy.

**Case Presentation:**

The TMC Library is unique in its multi-institutional user population, which presents distinct challenges in adopting a single sign-on authentication system. Our project involved OpenAthens technical support, information technology teams from six academic institutions, and over thirty publishers. Implementation included the creation of an OpenAthens parent account, an active user directory, a resource catalog, and installation of our OpenAthens credentials at each publisher site. Because the TMC Library serves multiple institutions, OpenAthens built a custom login page and a portal to support both single sign-on and a generic username and password option. This case report discusses the reasons why OpenAthens was chosen, the preparation methods for implementation, the various challenges encountered and resolved, and recommendations for other health sciences libraries considering this system.

**Conclusions:**

The OpenAthens system provides important benefits: granular usage reports, single sign-on access, and data to negotiate reduced pricing for online resources. With prior knowledge and preparation, health sciences libraries can successfully implement OpenAthens with customizations tailored to their specific resources and user population.

## BACKGROUND

Founded in 1915 and located in Houston, the Texas Medical Center (TMC) Library serves approximately 55,000 students, faculty, and researchers. We provide remote access to two major medical schools and four smaller academic institutions offering health sciences degree programs. Member institutions include the University of Texas Health Science Center at Houston, Baylor College of Medicine, Prairie View A&M College of Nursing, Texas Southern University College of Pharmacy and Health Sciences, Houston Community College Coleman College for Health Sciences, and the University of Texas MD Anderson Cancer Center.

IP-based authentication was the library's method for providing remote access to digital resources since first acquiring them for our collection. When the library moved to EZproxy in 2008, authentication via one IP address required all users to create library usernames and passwords. By 2013, the library's academic institutions began requesting that we adopt an authentication system that allowed single sign-on with institutional credentials and provided institutional usage statistics. Additionally, our EZproxy system, built upon an aging server infrastructure, did not provide reliable usage reports. Data harvesting from EZproxy logs took several days to run, often resulted in a crashed process, or simply failed to run at all. For these reasons, the TMC Library had strong incentives to choose OpenAthens.

The OpenAthens system is built on OpenID Connect, which allows user identity data to pass between an institution and a service provider (e.g., a publisher). As this service is maintained in a cloud-based environment, there are no local servers to maintain. OpenAthens's single sign-on (i.e., institutional user login) feature uses Security Assertion Markup Language (SAML) to identify authorized users at the service provider's platform. SAML communicates user identity attributes between an identity provider (i.e., the institution) and a service provider. Single sign-on means that patrons are no longer required to remember a separate set of credentials that will authorize access to their library's content. SAML technology appealed to our library in that “it allows the end-user to move from resource to resource without re-entering their credentials” [1].

OpenAthens also addresses our academic institutions' concerns about user privacy. The system employs a Lightweight Directory Access Protocol (LDAP) that stores patron usernames and passwords in an alphabetized directory that can be accessed by authentication tools, like proxy servers. It allows user credentials to be passed instantly to the publisher. This process occurs within seconds and is invisible to the user, thus providing them instantaneous access to resources [2].

Health sciences libraries need reliable authentication that brings authorized users to licensed content and that provides users with a seamless access experience. Here, we describe the TMC Library's efforts to meet these needs and its decision to adopt OpenAthens, which may resonate with other health sciences libraries who are considering a move to federated authentication to improve user experience and security.

## CASE PRESENTATION

Our implementation team consisted of staff from the library's Resource Management (RM) and Information Technology (IT) departments. The team conducted product comparisons between OCLC's cloud-based EZproxy system and Eduserv's OpenAthens. These were the only two vendors that could provide both single sign-on capability and usage reports for each of the library's member institutions. Vendors were graded in these key areas: initial customer service, product knowledge, product functionality, and responsiveness to customer concerns. EZproxy's vendor representatives were not responsive to our queries about subscription pricing, features, or services. This lack of information made it difficult to conduct a thorough comparison. However, a 2017 price comparison done by the previous IT manager showed that maintaining the old EZproxy system would have cost the library approximately $99,000 versus an estimated startup cost of $45,000 to implement OpenAthens. Based on the available information on price, functionality, service, and vendor responsiveness, we considered OpenAthens the more reliable choice.

After a product review, we then consulted health science organizations and libraries that had successfully implemented OpenAthens. Third Iron Technologies, our OpenAthens service provider, recommended we speak with Tom Waugh, senior officer at the Library Network Office for the US Veteran's Administration (VA). Since 2010, the VA has successfully used OpenAthens for user authentication for most of their hospital networks. In a phone conversation, Waugh reported that it was the VA that persuaded many STEM publishers to become OpenAthens compliant. Before gaining popularity in the United States, OpenAthens was first implemented by hospital networks within the United Kingdom's National Health Service. We also gained positive feedback about OpenAthens from email queries to the medical library listserv, MEDLIB. Julie Stielstra, library manager at Northwestern Medicine Central DuPage Hospital, stated that OpenAthens was a good solution: “We had been relying on IP authentication, but that was unreliable and frustrating for physicians in offices, people using their tablets, etc. The setup phase of course takes longest—you have to let them know every resource, database, journal, etc. for them to ‘Athenize’, and not EVERY journal can be. But it has been working nicely for nearly all our stuff. I'm very glad to have it” (email message to Joanne Romano, 2017 Mar 8).

Our team also reviewed OpenAthens's client testimonials, such as one given by Point Park University Library in Pennsylvania. The associate library director shared that Point Park “students don't even realize they're logging into anything different” [3].

We spent fourteen months on project planning and onboarding tasks. Below is a detailed description of our implementation process.

*January–June 2017:* The IT and RM department heads conducted a background investigation into EZproxy versus OpenAthens functionality. We met with Third Iron Technologies to discuss setting up the implementation of OpenAthens.

*July–December 2017:* The implementation team was formally established and conducted product comparisons as well as research into current OpenAthens customers' experiences. The team also reviewed the established membership of publishers in the OpenAthens federation.

*September–December 2017:* The implementation team collaborated with institutional IT departments to set up active directory access connections. To ensure minimal disruption of access, the team notified publishers of the upcoming authentication system change. Concurrently, team members also worked with OpenAthens tech support to set up the resource catalog.

*January–March 2018:* The implementation team informed library users of our move to OpenAthens via social media and website announcements. Our website included a link to the OpenAthens test portal so users could observe its functionality. The team presented this portal during a student mixer, enticing students to test with a prize drawing as an incentive.

*April 2018:* The TMC Library went live with OpenAthens.

*April–October 2018:* Implementation team members worked on resolving various technical challenges that arose after we went live with OpenAthens.

### Technical challenges

After we installed OpenAthens, there were a variety of technical challenges that had to be addressed. This required collaboration with the IT departments at each of our six member institutions. We worked with Eduserv and Third Iron to set up a phone conference with IT representatives from the institutions. Three institutions did not use single sign-on and had their users register with the TMC Library to create generic OpenAthens credentials. The other three used their own institutional credentials for single sign-on. This required them to provide Active Directory Federation Services integration profiles to establish subaccounts under the TMC Library's OpenAthens profile. Their IT departments also had to first conduct internal security reviews, which took several weeks to complete.

The OpenAthens system functions by using a redirector link that is added to a resource URL or by using a proxy link. The proxy link is used for publisher sites that are not part of the OpenAthens federation and therefore cannot recognize the redirector.

These new linking methods required the technical services team to expand their knowledge about linking syntax, URL encoding, and domain structure in the OpenAthens resource catalog. Team members reviewed technical sites such as the DOI system and OpenURL factsheet to gain more in-depth knowledge about URL coding [4]. For instance, we learned that the location of the auth path syntax (i.e., the characters that initiate the authentication action) within the URL affects linking behavior.

We also gleaned extensive knowledge from Third Iron Technologies, who were very instructive in helping us learn the OpenAthens linking mechanisms, particularly in how the redirector autodirects to an OpenAthens proxy URL when it recognizes a vendor link that is not OpenAthens compliant.

**Figure 1:**
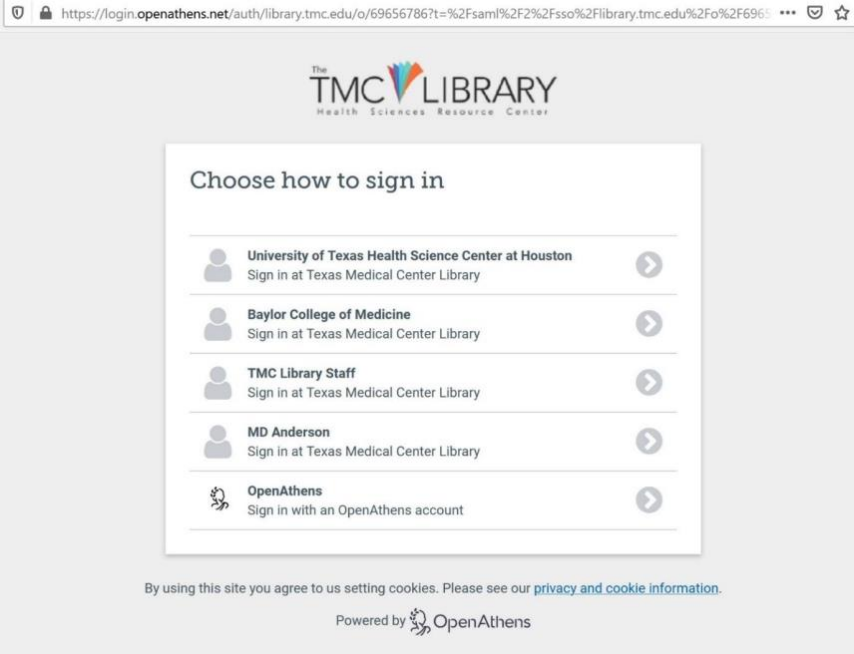
OpenAthens custom menu

**Figure 2:**
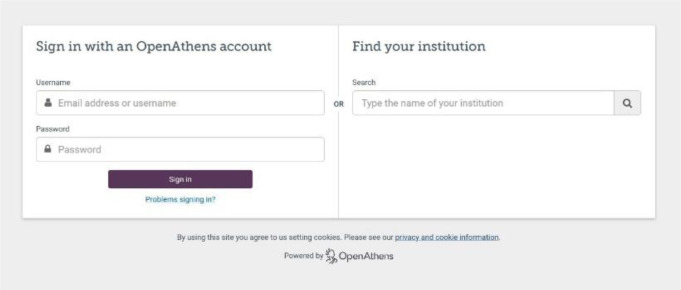
OpenAthens generic log-in page

To accommodate both credentialing methods, we had Eduserv build a custom OpenAthens sign-in page (Figure 1). This page required a drop-down menu that displayed all institutions' individual sign-in options. Building the custom sign-in page was a requirement for the library to finalize the OpenAthens license with Eduserv. This special feature also provided the Eduserv developers experience building custom sign-in pages for other library customers serving multiple institutions. Clicking on the OpenAthens menu choice takes the user to the log in page (Figure 2).

Aside from technical challenges on our side, we also encountered problems with various publisher sites. The challenges of activating OpenAthens on publisher sites were well documented in Michelle Kraft's review of the product [5]. We agreed with Kraft's assertion that this one-time task was cumbersome since it required connecting with multiple publishers and technical support contacts.

Working with publisher technical support was indeed a barrier to the onboarding process. While some publishers allowed our library staff to manually add the OpenAthens credentials to their platforms, other publishers did not. Some took as long as two weeks to complete this task, which created project delays. This necessitated persistent email requests every few days to lagging tech support contacts until all activation tasks were completed.

For months following the end of the project, library staff also discovered that some of our journal publishers (e.g., Slack, PNAS, American College of Physicians, Mary Ann Liebert) were dropping our OpenAthens credentials and reverting to our EZproxy IP range without prior notice. Staff discovered these errors only after receiving user complaints of access denials.

We also encountered problems with vendor platforms (e.g., SAGE, MaryAnn Liebert, Future Medicine) using security layers that did not work with federated authentication. In particular, the CAPTCHA challenge initiated at Atypon-supported platforms prevented the authentication process from occurring at the vendor site.

These and other publisher issues with OpenAthens are well documented. Arsenault et al. reported that “not all publishers and platforms support OpenAthens, resulting in librarians having to manage multiple authentication workflows simultaneously. There are also occasional Digital Object Identifier (DOI) problems where some DOIs will not resolve for OpenAthens, leaving the content inaccessible” [6].

The ability to authenticate by going directly to the vendor platform is considered a benefit of federated systems. However, we discovered that publishers display the OpenAthens option in random areas on their websites, with some in very nonintuitive locations. The design inconsistencies resulted in a poor user experience when authenticating to full text. Only through trial and error, such as clicking on PDF icons, “Get Access” buttons, or other access indicators, would users chance upon the vendor's OpenAthens authentication option. We therefore added instructions about the various login options at vendor sites to our existing user education documentation.

### Project management challenges

The library's RM and IT departments were responsible for the setup and implementation of OpenAthens as well as troubleshooting vendor platform and access issues. While the OpenAthens onboarding tasks were being coordinated, the library was already committed to a new content management/discovery portal migration with the Texas Health Science Libraries Consortium (THSLC). The THSLC's decision to migrate to Alma/Primo VE coincided with our move to OpenAthens. After our implementation team began its preliminary work, the Alma/Primo VE training schedule was released. As a result, our library staff had to simultaneously learn the Alma/Primo VE system while onboarding with OpenAthens.

Moving to Alma/Primo VE added an element of linking complexity for managing resources in OpenAthens. Alma uses “parsers,” special linking scripts that create dynamic links between the content management system and the Primo VE discovery portal. This required multiple variations of the OpenAthens linking syntax for successful authentication and access to certain resources.

Library administration had a mandate to move forward with these multiple system migrations. Managing workflows to adopt a new authentication system as well as a new content management/discovery portal system made it difficult for the implementation team to provide adequate OpenAthens training for the library staff. Not surprisingly, this also made it difficult for staff to adopt OpenAthens. The insufficient opportunity to prepare staff for the migration, coupled with the many access issues encountered, created roadblocks to project success.

The TMC Library did not have direct communication channels with its member institutions, which created significant barriers to user education. For example, it was difficult for library staff to notify all users to replace their bookmarked EZproxy URLs with the OpenAthens redirector URL. For many months after the migration, some users continued to try and access resources with the obsolete EZproxy linking mechanism, which engendered high frustration with the library. To circumvent these challenges, staff created detailed video tutorials and LibGuides, which were advertised and made accessible on the library's website. These educational materials clarified the two different methods of OpenAthens authentication (generic or institutional single sign-on) for users. The videos provided step-by-step instruction on how to log in to Open Athens. The LibGuides offered further instruction by showing users how to register and set up a generic OpenAthens account.

### Post-migration progress

Despite these challenges, we were eventually able to provide a user-friendly authentication path with OpenAthens.

To resolve the CAPTCHA messages presented by Atypon, we gained direct access to their product development team and had them work with Eduserv to remove that security layer. This was unprecedented because as a third-party platform provider, Atypon does not typically communicate or collaborate with libraries.

We improved staff communication by sending weekly email updates about ongoing and resolved vendor access problems.

During this project, one of our key member institutions, Baylor College of Medicine, developed a TMC Library page on their intranet. This page now provides an internal communication portal for the library to share information on resource access issues, library services, or other relevant library news.

## DISCUSSION

Our reasons for choosing OpenAthens included secure authentication, user privacy, a seamless experience, and granular usage statistics at the user level that could not be harvested from vendor platforms. OpenAthens was chosen by other libraries seeking similar solutions. For instance, UNC-Charlotte found that as every resource use must pass through the OpenAthens proxy server, it captures more precise statistics per user, regardless of their location on or off campus [7].

User privacy was a real concern for us in evaluating OpenAthens. The institutions we serve include a mix of academic research institutions and one teaching hospital, so protecting user data is a crucial issue. OpenAthens's LDAP connector allows our system to connect directly to the institutions' existing LDAP servers, where users' local accounts are maintained by their home institutions under their own standard security settings [8]. User accounts can be deactivated immediately when employees and students leave their institution. This key feature was an important selling point for the library.

OpenAthens's reliability in capturing usage data at the institutional level was another integral benefit for our library. We have seen vast improvements in the reporting capabilities. To date, there have been no delays in report generation, crashed processes, or data gaps when harvesting usage reports. These reports have unexpectedly proven to be a valuable negotiation tool with our vendors. Typically, our library pays for remote access to all six member institutions regardless of whether they use our resources or not. The OpenAthens usage reports revealed that three institutions displayed zero to fewer than ten clicks to certain resources over a fourteen-month period, which we reported to the providers. As a result, we were able to negotiate down the subscription fees, paying only for those institutions that were actually using the resources. For example, upon sharing the usage data, we kept our renewal fees flat for the Radiological Society of North America, *Annals of Internal Medicine*, and others for the 2019–2020 fiscal year.

Roger Schonfeld noted that because SAML-based systems like OpenAthens can capture usage patterns for specific patrons and their institutions, customer libraries might use that information to leverage publisher pricing and thus gain higher value for their library resources [9].

The use of OpenAthens data as a collection development tool and a measure of library impact was not a benefit factored into our purchase decision. However, after researching the literature, we learned that some OpenAthens customers were considering its use to measure the impact of library collections on student success. For example, Leeds Beckett University plans an in-depth investigation of OpenAthens usage metrics, to assess library impact on student learning [10]. This unexpected benefit is one that we want to explore in the future.

Reflecting on this experience, it was clear that we needed to increase our project management skills at the TMC Library. Better planning before the OpenAthens migration could have helped us avoid some of the roadblocks we encountered. Regarding the mandate to handle multiple new system migrations at the same time, we should have stressed to institutional administrators the potential negative impact on users and library staff. Had the option existed, we would have put the OpenAthens project on hold until Alma/PrimoVE was fully implemented and staff/user training goals met. Before either project began, we should have taken more time to build effective communication channels—both internally with staff and externally with our academic partners. Having these in place ahead of time, we could have kept users and staff better informed about our progress. This may have also mitigated staff and user frustration from the many glitches and access disruptions that occurred during implementation.

We ultimately realized that our choice of Third Iron over EBSCO as an implementation partner put more of the legwork on our implementation team to manage the OpenAthens onboarding tasks. It was the team's responsibility to create the project plan, set up the OpenAthens catalog, obtain active user directories from the IT departments, contact vendors to swap out EZproxy IP ranges for OpenAthens credentials, follow up with vendors to resolve authentication issues, and develop user education and marketing materials about OpenAthens. Case studies from other libraries revealed that they received assistance from EBSCO in setting up project planning, tracking spreadsheets, contacting vendors, and other tasks. Third Iron was an excellent liaison between Eduserv and the TMC Library, but we chose to be responsible for the bulk of the labor required.

However, we benefitted greatly from Third Iron's early partnership with Eduserv in the development of OpenAthens. It was Third Iron that encouraged Eduserv to develop the OpenAthens proxy URL for nonfederated publishers. Because of their technical expertise, Third Iron was able to clarify our understanding of SAML-based authentication, linking syntax, coding, the OpenAthens Redirector, the OpenAthens proxy URL functionality with service providers, and the mechanics of URL generation to resolve to full text articles. This intensive education was extremely valuable for our team members and, by extension, our users. Partnering with Third Iron made the TMC Library quite knowledgeable and independent in managing its OpenAthens system.

At the time of this writing, our users seem satisfied with OpenAthens. A review of our access issues tickets from January 2019 to January 2021 found that access issues were typically the result of vendor platform updates, platform migrations, or vendor entitlements not being properly set up during renewal periods. OpenAthens itself was not the cause for access disruptions.

Based on our library's experiences, we recommend several pre- and post-migration activities that may help other health sciences libraries achieve a successful migration. Before these activities begin and well before the “go live” date to roll out the new system, libraries should develop an OpenAthens migration checklist.

- Allow for a minimum twelve-month time window before you begin any project planning and onboarding activities.
- Identify appropriate publisher technical support contacts and be sure to notify them of the changes needed at least two weeks before the migration process begins. This will confirm that you have their current contact information before email requests need to be sent. Give them a firm due date as to when you need OpenAthens credentials added to your administrative profiles. Follow up with emails within forty-eight hours of your initial email requests.
- Create clear instructions and messages for end users and disseminate widely using the library website, blogposts, social media, and LibGuides. Contact institutional administrators who can tap into academic communication channels that will allow you to effectively educate all users about the new authentication system.
- Include staff with appropriate expertise on your implementation team and have them help develop the migration checklist. Assign one individual to coordinate all communications to users and library staff.
- Conduct user testing in an OpenAthens sandbox environment prior to actual migration. Send out user satisfaction surveys before and after the new system is rolled out. Delegate team members to collect, document, and distribute the survey responses. Armed with this feedback, the implementation team can promptly address OpenAthens technical or educational challenges for users as appropriate.
- Schedule weekly meetings between the implementation team and library staff to gather internal feedback, discuss concerns, and proactively resolve issues if possible. Ensure that resource access is uninterrupted by running the old and new authentication systems simultaneously if possible. Redirect old authentication links to the new system URL. Tell your users to update their bookmarked authentication links well in advance of the “go live” date. Ensure that your library branding is displayed on all publisher websites before and after going live with OpenAthens.

Migrating to a new federated authentication system presented multiple challenges for our library staff. There were unexpected technical issues with our academic partners and our publishers that required creative solutions. Our internal and external communication channels presented their own problems. Despite these challenges, we successfully implemented OpenAthens. Furthermore, these experiences provided the implementation team and library staff with valuable lessons moving forward.

At the start of our research in December 2019, finding comparable OpenAthens experiences was difficult. As noted previously, ours is a stand-alone library serving six different academic institutions, and we wanted to find stories from libraries who also serve various user groups at multiple locations. Since 2019, however, we have uncovered several articles from libraries serving multiple campuses that report similar challenges moving from EZproxy to OpenAthens. After reviewing case studies from UNC-Charlotte and Eastern Carolina University libraries, Arsenault et al. report that while federated authentication systems are not perfect, they do eliminate many access issues that occur with IP-based recognition [6]. We agree with this assessment.

Robust project planning, thorough staff/user education, and consistent communication are key to ensuring a smooth migration with few access disruptions. Today, the TMC Library provides single sign-on authentication, usage statistics for six academic stakeholders, data to negotiate reduced pricing for online resources, and a positive user experience to accessing full text.

Our future plans include the distribution of a new survey to verify current user satisfaction with OpenAthens and examination of resource usage by user categories to augment collection development analysis. Given the growing shift toward federated authentication, as evidenced by the 2019 ResourceAccess21 project, more health sciences libraries will be seeking best practices for adopting a federated solution. It is notable that Lisa Hinchliffe discussed the potential loss of *any* option for IP-based authentication that many libraries still rely upon today [11]. Considering these developments, we hope that our library's experience will benefit other health sciences libraries considering a move to OpenAthens.

## Data Availability

Data associated with this article are available in the TMC Library's institutional repository at https://digitalcommons.library.tmc.edu/library_docs/26.
